# Moderate Effect of Flavonoids on Vascular and Renal Function in Spontaneously Hypertensive Rats

**DOI:** 10.3390/nu10081107

**Published:** 2018-08-16

**Authors:** María D. Paredes, Paola Romecín, Noemí M. Atucha, Francisco O’Valle, Julián Castillo, María Clara Ortiz, Joaquín García-Estañ

**Affiliations:** 1 Department of Physiology, School of Medicine & Biosanitary Research Murcian Institute (IMIB), University of Murcia, 30120 Murcia, Spain; madopaca@um.es (M.D.P.); paodunromec@gmail.com (P.R.); ntma@um.es (N.M.A.); clara@um.es (M.C.O.); 2 Department of Pathological Anatomy, School of Medicine, IBIMER (CIBM) & Ibs.GRANADA, University of Granada, 18016 Granada, Spain; fovalle@ugr.es; 3 Institute of Aging & R & D, Nutrafur SA-FRUTAROM Group, 30820 Alcantarilla, Spain; j.castillo@nutrafur.com

**Keywords:** flavonoids, nitric oxide, heart, kidney, sodium balance, phenylephrine, acetylcholine

## Abstract

Many studies have shown that flavonoids are effective as antihypertensive drugs in arterial hypertension. In the present work, we have analyzed the effects of some flavonoid extracts in the spontaneous hypertensive rat model (SHR). An important feature of this study is that we have used a low dose, far from those that are usually applied in human therapy or experimental animals, a dose that responded to the criterion of a potential future commercial use in human subjects. Treatments were carried out for 6 and 12 weeks in two groups of SHR rats, which received apigenin, lemon extract, grapefruit + bitter orange (GBO) extracts, and cocoa extract. Captopril was used as a positive control in the SHR group treated for 6 weeks (SHR6) and Diosmin was used as the industry reference in the SHR group treated for 12 weeks (SHR12). Captopril and GBO extracts lowered the high arterial pressure of the SHR6 animals, but none of the extracts were effective in the SHR12 group. Apigenin, lemon extract (LE), GBO, and captopril also improved aortic vascular relaxation and increased plasma and urinary excretion of nitrites, but only in the SHR6 group. Kidney and urinary thiobarbituric acid reactive substances (TBARS) were also significantly reduced by GBO in the SHR6 rats. Apigenin also improved vascular relaxation in the SHR12 group and all the flavonoids studied reduced urinary thiobarbituric acid reactive substances (TBARS) excretion and proteinuria. Vascular abnormalities, such as lumen/wall ratio in heart arteries and thoracic aorta, were moderately improved by these treatments in the SHR6 group. In conclusion, the flavonoid-rich extracts included in this study, especially apigenin, LE and GBO improved vascular vasodilatory function of young adult SHRs but only the GBO-treated rats benefited from a reduction in blood pressure. These extracts may be used as functional food ingredients with a moderate therapeutic benefit, especially in the early phases of arterial hypertension.

## 1. Introduction

Experimental studies with foods that are rich in flavonoids are an interesting area of research to understand the pathophysiology of the mechanisms underlying their blood pressure lowering effects during arterial hypertension [1,2]. Thus, some studies have linked the elevated flavonoid-rich consumption with a reduced cardiovascular risk [3,4]. Moreover, the use of natural products with lower side-effects is an interesting possibility to be considered when treating several pathologies [5]. Several studies have correlated the consumption of flavonoid-rich food as well as isolated compounds, with the beneficial effects on several cardiovascular indices, such as vasodilation, arterial pressure, and cardiovascular risk markers [6,7]. Of interest, many flavonoids increase the bioavailability of endothelial vasodilator factors, mainly nitric oxide (NO) and also endothelium-derived hyperpolarizing factor (EDHF), and inhibit the production of pro-inflammatory substances, which considerably improves the function of vascular endothelium [8,9]. They affect also several renal factors that promote diuresis and natriuresis, which may contribute to their well-known antihypertensive effect [10]. 

In a previous study in the L-NAME (nitric oxide-deficient) model of arterial hypertension [10], we showed that some flavonoids, especially apigenin, were effective in reducing the elevated blood pressure that is associated with the chronic deficiency of nitric oxide (NO). An important feature of that study [11] is that we used a low dose, which was far from those usually applied in human therapy or experimental animals [12,13], a dose that responded to the criterion of a possible future commercial use in human subjects. As in other studies [14,15], the antihypertensive effects of these flavonoids were due to a mixture of vasodilator and antioxidant effects [11].

In the present study, we have carried out a similar study in another model of arterial hypertension, the spontaneously hypertensive rat (SHR), thought to be the most comparable with the human form of hypertension [16,17]. SHR rats have increased activity of fluid retention mechanisms, sodium reabsorption, and increased vascular resistance. This increase in vascular resistance being first produced by neurogenic mechanisms and later by structural vascular changes [18,19,20]. Therefore, in the present study, we have evaluated the vascular and kidney effects of some flavonoid extracts in SHR rats.

## 2. Material and Methods

### 2.1. Animals

The experiments were carried out in male SHR and their control WKY rats (Wistar Kyoto rats, Harlan Lab, Barcelona, Spain) housed in a temperature controlled environment, with 12 h:12 h (light:dark) cycle in the Animal Care Facility of the University of Murcia (REGAES300305440012). The animals were maintained according to the guides that were established by the European Union for protection of the animals (86/609/EEC). The experimental procedures were approved by the Animal Care and Use Committee of the University of Murcia (C1310050303). 

### 2.2. Experimental Groups

There were two treatment groups of male SHR, one treated for six weeks and another group treated for 12 weeks, along with their respective controls (WKY) rats. 

SHR6. The first treatment group (six weeks) was composed of 56 SHR (8–9 week old, initial weight, 185–271 g) and six WKY rats (initial weight, 213–220 g). This group was composed of the following experimental groups:

(1) Control (*n* = 6), WKY rats without any treatment.

(2) SHR (*n* = 7), SHR rats without any treatment.

(3) Apigenin (A, *n* = 6), SHR rats treated with A (1.44 mg/Kg/day).

(4) Lemon extract (LE, *n* = 7), SHR rats treated with LE (2.84 mg/Kg/day).

(5) Grapefruit + Bitter Orange Extracts (GBO, *n* = 7), SHR rats treated with GBO extract (9.28 mg/Kg/day).

(6) Cocoa extract (COE, *n* = 6), SHR rats treated with COE (2.52 mg/Kg/day).

(7) Captopril (CPT, *n* = 6), SHR rats treated with CPT (100 mg/Kg/day). 

This angiotensin converting enzyme inhibitor was used as a positive control, since it has been shown to be very effective for lowering blood pressure of the SHR model (18–19).

SHR12. The second treatment group (12 weeks) was composed of 38 SHR (8–9 week old male, initial weight, 210–267 g) and normotensive Wistar-Kyoto (WKY) rats (initial weight, 212–230 g). This was composed of seven experimental groups, which were similarly treated as the SHR6 groups, except for the captopril group, which was changed to Diosmin (D, 7.16 mg/Kg/day). Since the antihypertensive properties of this angiotensin converting enzyme inhibitor in the SHR rats have been studied extensively and its ability to control blood pressure chronically has been very well documented [21,22,23], we decided to use another flavonoid in this SHR12 group. In this case, a well-known reference drug in the industry. Diosmin is probably one of the flavonoids that was most used in diverse products directed to the cardiovascular system health [24]. Also, it is a flavonoid of citric origin, such as some of the other flavonoids used. All of the groups were composed of six rats, except the SHR untreated group, with nine. 

The doses and composition of the different extracts have been previously published [11]. Briefly, the composition of the different extracts that were used in this study was determined by High-Performance Liquid Chromatography (HPLC) and in all the extracts and purified compounds assayed, the unique active components are flavonoids [11] (Table 1).

The doses were selected to be economically competitive in the case of future commercial use. The projected cost was approximately 0.04€ per dose per day. Doses were calculated by taking into account the recommended daily dosage and the commercial price of the compounds [11].

All of treatments were administered in the drinking water during the required time, 6 or 12 weeks, except for Diosmin, which was given orally, in powder feeders (Tecniplast, Radnor, PA, USA), mixed with the powdered food. The doses of the extracts in the drinking water were adjusted every day taking into account the amount ingested the day before. The animals had complete access to a standard rat formula with a 0.5% of sodium content (104 mEq/Kg) and tap water, with or without treatments. All products, except L-NAME and captopril (Sigma, Madrid, Spain, were kindly provided by Nutrafur SA-FRUTAROM Group.

### 2.3. Experimental Procedures

All of the procedures are the same as those previously published [11] in our paper with L-NAME hypertensive rats. Briefly, metabolic data (water intake, diuresis, and natriuresis) were obtained by housing the animals in individual metabolic cages. Blood pressure was measured under pentobarbital anesthesia by using an indwelling catheter in the femoral artery. Blood was also collected through this catheter and plasma that was obtained after centrifugation. All of the samples were frozen at −80 °C. Finally, the kidneys, the heart and the abdominal aorta were removed for later studies. Vascular reactivity was carried out with aortic rings in individual organ baths, and the protocol was exactly the same as the one described previously [11]. In brief, dose–response curves to phenylephrine (PHE) to analyze vasoconstrictor response, and the vasodilator responses were evaluated with acetylcholine (ACH) before and after addition of the nitric oxide synthase inhibitor L-NAME and with sodium nitroprusside (SNP) to test the direct vasodilator responses and the smooth muscle functionality. TBARS (thiobarbituric acid reactive substances) in plasma and kidney were determined as a measure of lipid peroxidation by using a colorimetric method described previously [11]. The protein concentration was measured with a bicinchoninic acid based-method (Sigma, Madrid, Spain, and plasma and urinary excretion of nitrites was determined by the Griess reaction. The histological analysis was performed after the aortic, cardiac, and renal tissue samples were fixed in 10% buffered formaldehyde, embedded in paraffin, and sectioned (4 µm), as previously reported [11,25,26,27].

### 2.4. Statistical Methods

Data are shown as the mean ± standard error. Differences between groups were compared by one-way ANOVA, followed by Duncan’s multiple range test as the post hoc test. The values of EC_50_ (effective concentration at 50% of the maximum response) were calculated from the individual dose-response curves and expressed as the negative logarithm (pEC_50_). Differences were considered statistically significant at a *p* level lower than 0.05.

## 3. Results

### 3.1. Blood Pressure and Urinary Variables

**Figure 1:**
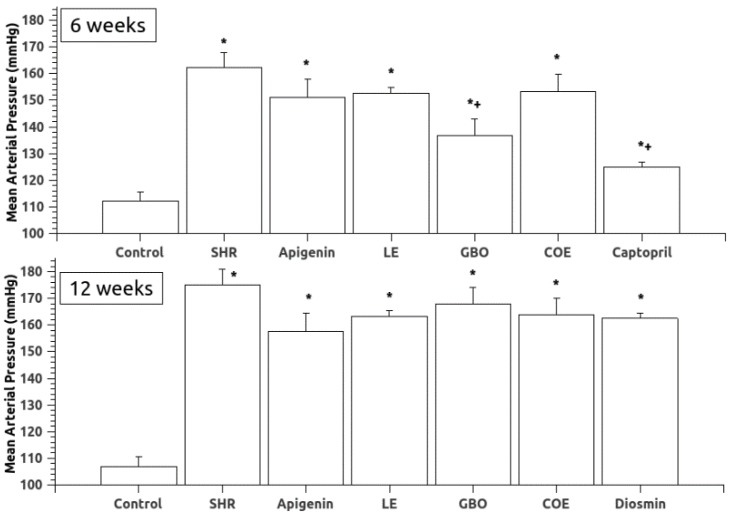
Mean arterial pressure (MAP) in the experimental groups. SHR (spontaneously hypertensive rats), LE (lemon extract), GBO (grapefruit + bitter orange extract), COE (cocoa extract). Data are mean ± S.E.M. * *p* < 0.05 vs. WKY; + *p* < 0.05 vs. SHR.

Mean arterial pressure (MAP) data are shown in Figure 1 and Table 2. In the SHR6 group, most of the treated groups showed slightly lower MAP values than the group with untreated hypertension (SHR), but a significant difference was found only in the case of the treatment with GBO and CPT. In the SHR12 group, no statistically significant differences were observed in the treatment groups as compared to the SHR untreated rats. Heart rate was significantly decreased by captopril in the SHR6 group and GBO, COE, and D-treated groups showed lower heart rate values in the SHR 12 group.

Table 3 lists some metabolic parameters of all the experimental groups. In the SHR6 group, no significant differences in body weight were observed among the experimental groups. In the SHR12 group, the group that received GBO showed a significantly lower body weight when compared with the untreated rats. Diuresis was not statistically different in the experimental group SHR6, except for the group that was treated with captopril. In the SHR12 group, diuresis was significantly lower only in COE and D-treated groups, as compared with the untreated SHR group (Table 3). Regarding sodium balance, there were no differences between untreated WKY and SHR rats, but the SHR6 group that was treated with LE and captopril had lower sodium balances than the untreated groups. In the SHR12 groups, a greater sodium balance was observed in the GBO and COE-treated groups (Table 3).

### 3.2. Vascular Function

**Figure 2:**
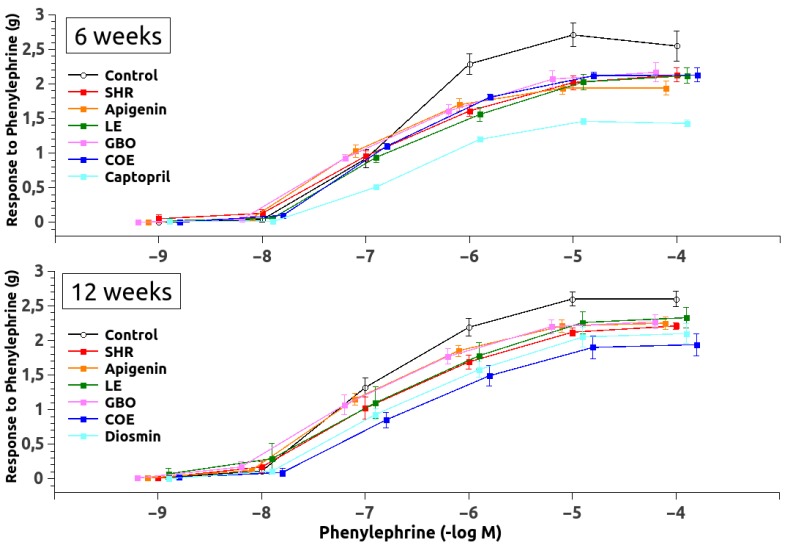
Pressor response to phenylephrine in aortic rings. Abbreviations: SHR (spontaneously hypertensive rats), LE (lemon extract), GBO (Grapefruit + bitter orange extract), and COE (cocoa extract).

Dose-response curve to PHE was significantly shifted downwards in the arteries from all of the treated and untreated groups (Figure 2), reducing the maximum contractile responses in all of the SHR6 groups, as compared to the controls. The pEC_50_ was significantly enhanced also in all SHR6 groups and none of the treatments changed them (Table 4). In the SHR12 groups, the maximum contractile responses were also reduced compared with the controls response, but the pEC_50_ did not change significantly (Table 4). 

**Figure 3:**
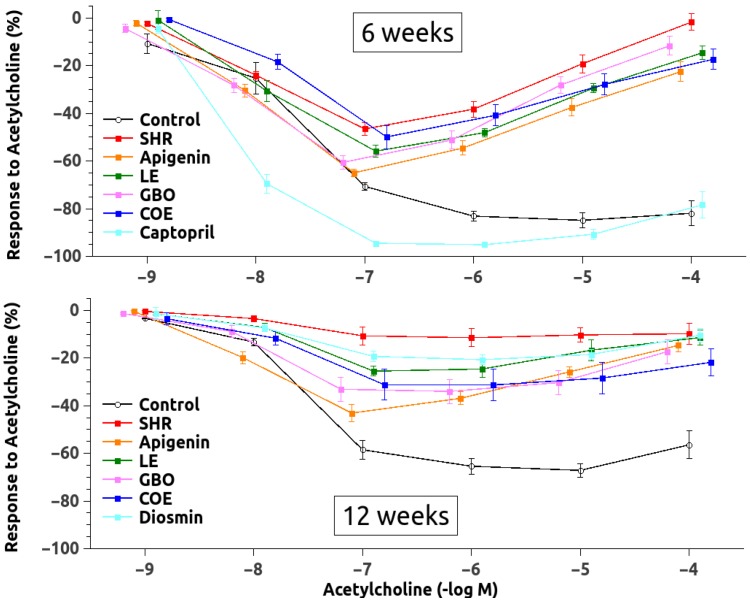
Vasodilatory response to acetylcholine in phenylephrine-preconstricted aortic rings. Abbreviations: SHR (spontaneously hypertensive rats), LE (lemon extract), GBO (grapefruit + bitter orange extract), and COE (cocoa extract).

Maximal ACH-induced vasodilatation (Figure 3 and Table 4) was significantly blunted in aortic rings from the SHR6 rats when compared to control rats. The ACH relaxation improved significantly in the aorta from the SHR6 rats that were treated with A, LE, and GBO, although the relaxation remained lower than in the control rats, except for the captopril-treated group, which was completely normalized. After administration of acute L-NAME (10^−4^ mol/L) to these aortic rings, the relaxant responses in response to ACH were further reduced, and almost abolished, but there were some residual and valuable responses only in the captopril-treated group. Regarding the SHR12 group, a similar decrease was observed in all of the groups, and only the Diosmin-treated group showed a significantly greater relaxation (Table 4). Vasorelaxation in response to SNP was significantly reduced in the flavonoid-untreated SHR6 and SHR12 groups when compared with the control rats. SNP-induced vasorelaxation improved significantly in all flavonoid-treated groups (Table 4). 

### 3.3. Effect of Flavonoid Extracts on Oxidative Stress Status

Values of TBARS, nitrite and urinary protein excretion are shown in Table 5. TBARS significantly increased only in the plasma and urine of the untreated animals as compared with controls. In the SHR6 group, only captopril reduced plasma TBARS, whereas captopril, LE, GBO, and COE reduced renal levels when compared to the untreated SHR group. In the SHR12, only apigenin reduced renal TBARS values, although the urinary values were significantly lower in all of the flavonoid-treated SHR12 rats. Regarding nitrite urinary excretion, only treatment with captopril in the SHR6 and GBO in the SHR12 groups showed a significantly greater value as compared to the untreated group. Also, urinary protein excretion was significantly higher in the untreated group, but proteinuria was reduced in the LE-treated group (in SHR6 and SHR12) and in the groups that were treated with GBO, COE, and D (in SHR12).

### 3.4. Histopathology Results

**Figure 4:**
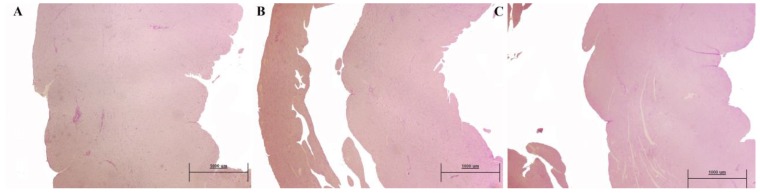
Representative images of modification in interventricular heart septum thickness. (**A**) from a SHR untreated rat of the SHR6 group; (**B**) from a WKY rat of the SHR6 group; and, (**C**) from a captopril-treated rat of the SHR6 group. Bar: 100 micrometers (periodic acid-Schiff stain (PAS), original magnification × 20).

The analysis of the heart revealed that both SHR untreated and treated groups (both SHR6 and SHR12) showed no infarcts, hyaline arteriopathy, or fibrinoid necrosis (data not shown), features that were observed in the study performed in the L-NAME hypertension [11]. Wall-lumen ratio of coronary arteries was decreased in the SHR6 when compared with control and treatments with A and COE increased them. In the SHR12 group, there were no changes. Interventricular heart septum thickness was significantly higher in the SHR6 untreated group when compared to the control rats and only captopril lowered to a normal level. In the SHR12 group, the values of all the experimental groups were very similar without significant differences between them (Figure 4).

**Figure 5:**
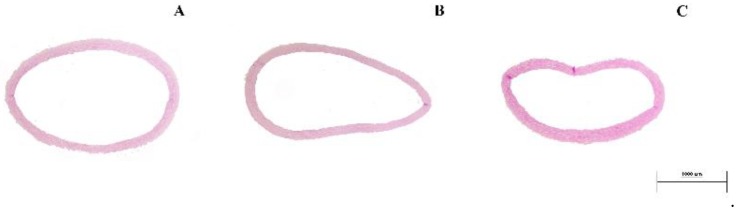
Representative microphotograph of thoracic aorta wall thickness modification in SHR12 rats. (**A**) flavonoid-treated group (COE group); (**B**) WKY control rat; and, (**C**) SHR control rat. Bar: 100 micrometers (PAS, original magnification × 0.2).

**Figure 6:**
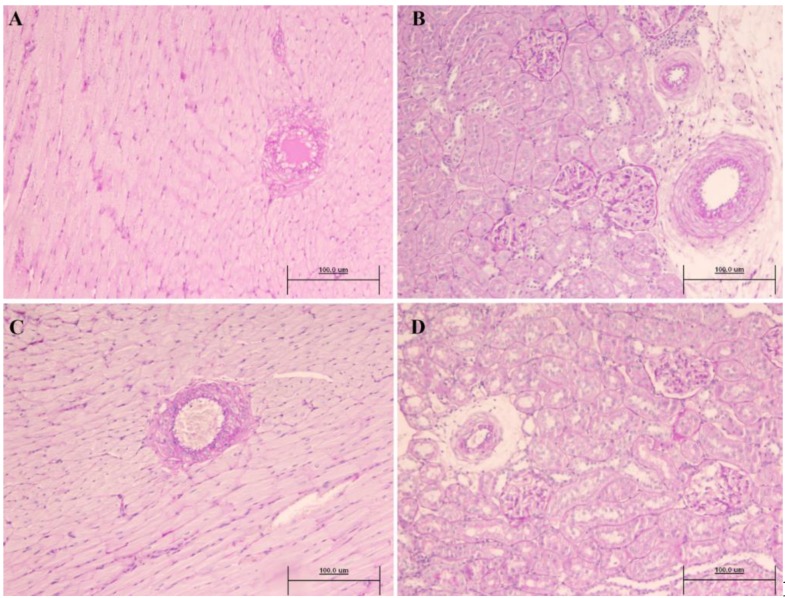
Absence of heart parenchyma damage, and no tubulointerstitial, glomerular or vascular kidney lesions in in SHR6 ((**A**) and (**B**)) and SHR12 ((**C**) and (**D**)). Bar: 100 micrometers (PAS, original magnification × 20).

With respect to the thickness of the abdominal and thoracic aorta (Table 6), there were significant reductions in the thickness of the abdominal aorta in all of the SHR6-treated groups only. In the SHR12 group, the tendency of the flavonoid-treated groups was to show an increase in the thickness in both vessels (Figure 5). Regarding the kidney (Table 6), significant increases were found in the LWR (lumen to wall ratio) parameter in the LE and GBO-treated SHR6 groups. Also, the absence of hyaline arteriopathy (HA) and tubular cylinders (TC) was evident in all of the treated SHR6 groups, except in the A group. In the SHR12 groups, HA and TC were also found in the untreated SHR and in the groups treated with A and COE (Figure 6).

## 4. Discussion

The results of this study show that some of the flavonoids studied have a very modest effect on blood pressure and renal and vascular function in the SHR model of arterial hypertension. Moreover, these effects can be observed only in the younger animals, treated for six weeks. Doubling the treatment time (12 weeks) almost eliminated these beneficial effects.

In the SHR6 groups, only GBO had a significant effect on arterial pressure and this was accompanied by a significant improvement of the vascular vasodilator response, which is likely due to an increased production of vascular NO, as indicated by a higher plasma nitrite concentration. A reduction in renal oxidative status with beneficial changes in the histopathological parameters in heart and kidney was also observed in this group. Although blood pressure was not significantly modified in the apigenin and lemon extract-treated groups, similar vascular and renal changes were observed in these animals when compared to the GBO-treated group. 

As reported previously [11], the dose chosen of each of the treatments responds to the objective criterion of potential future application in humans. Therefore, the doses that were ingested daily by our animals are very low compared to those used in other studies with similar compounds, since generally, in animal studies, doses much higher are used, proportionally, than those applied in a human therapy [5,15].

It is clear that both types of arterial hypertension models are very different. It seems that the flavonoids that we have used are more useful in the nitric oxide-deficient hypertensive animals. In fact, one of the most universal effects of flavonoids is through the augmentation of NO synthesis and action [14,15]. In this way, they can be of interest in diseases of endothelial dysfunction [28]. However, the SHR model is a completely different model, with an important genetic component [20]. Hypertension in the SHR model is the result of elevated peripheral vascular resistance, which is produced first by neural and kidney factors, with structural vascular changes due to elevated vascular protein synthesis occurring later as a consequence of the chronic elevation in blood pressure. As observed, our results clearly confirm that the inhibition of the renin-angiotensin system is one of the treatments of choice for this model and this has been shown previously [20,21,22,23]. Although we only used captopril in the SHR6 group, it is likely that the chronic antihypertensive effect could be also observed in the SHR12 group, as it has been demonstrated previously [20,21,22,23]. However, it is true that a positive control better than Diosmin would have been beneficial for the study.

Many studies have described the antihypertensive effect that is related with the consumption of flavonoid-rich products. Thus, in vitro studies have found that flavonoids such as genistein, quercetin, and epicatechin regulate (directly or indirectly) NO production in isolated vessels or cultured endothelial cells [8,29,30,31,32]. In our data, A, LE, GBO, and, of course, captopril, improved the acetylcholine relaxation in the SHR6 rats, which is dependent on NO production (after acetylcholine administration). Moreover, in these SHR6 animals, the administration of sodium nitroprusside also improved the reduced relaxation in the animals receiving all of the flavonoids, which is indicative of an action that is close to the NO effector in the smooth muscle cell, at the level of cGMP. These data also speak in favor of a reduced production of vascular NO in the SHR model, as suggested previously [20].

Some other effects have been suggested to explain the increased endothelial NO bioavailability that is promoted by flavonoids. In fact, a regular consumption of flavonoids can significantly improve the oxidative status as well the endothelial function [8,11,15,28,33]. In the present study, we detected a significant increase in reactive oxygen species levels, as measured as TBARS, in plasma and urine in the SHR untreated animals. It is likely that the reduction in kidney TBARS, as observed in some of the flavonoid-treated groups (Table 5), is also contributing to the normalization of BP. It may be interesting to consider that the treatments with a greater specific antioxidant efficacy are those having flavonoids with B-ring catechol structure (3′,4′-dihydroxy), LE (eriocitrin), and COE (catechin compounds). 

It is known that the chronic elevation of systemic blood pressure is associated with the presence of proteinuria and the development of glomerulosclerosis [34,35], as our data show (Table 5). The flavonoids treatments showed a reduction in proteinuria, but it only reached a significant difference in the LE-treated SHR6 group. Interestingly, proteinuria was also significantly reduced in the SHR 12 groups, and this is a result that merits further study.

The metabolic and hemodynamic changes of hypertension are also associated with the development of structural abnormalities, such as left ventricular hypertrophy, cardiac fibrosis, necrosis and protein remodeling, as well as with vascular wall hypertrophy [36,37], some of them shown in the present results. Maybe because of NO deficiency and probably also because of the hypertensive load on vascular tissues, increased monocyte and platelet adhesion with the release of growth factors would contribute to the thickening of the vascular wall. The proliferation was limited to the media, which agrees with the findings of others [33,34,35]. All of the flavonoids treatments were effective to reduce the abdominal aortic thickness in the SHR6 group, but only captopril reduced cardiac hypertrophy. Regarding the renal structural changes, only in the SHR12 group, flavonoids seem to exert some beneficial effect, since hyaline arteriopathy was only present in the SHR untreated group, and tubule cylinders were not observed in the LE, GBO, and Diosmin-treated groups.

Our results suggest that the flavonoids included in this study, and are already present in the market as nutritional supplements, may be used as functional food ingredients, but the beneficial effect on hypertension is moderate, especially in the younger SHR group. Further studies are necessary to elucidate the mechanisms involved in their effects. In any case, our results suggest that the effects of these flavonoids may be related to a combination of vasodilator and antioxidant actions.

## 5. Clinical Perspectives.

Flavonoids are important substances with biological actions of interest in arterial hypertension. We aimed to analyze the role of some flavonoids in a model of arterial hypertension, the spontaneously hypertensive rat (SHR), thought to be the most comparable with the human form of hypertension. 

Grapefruit extract significantly reduced the elevated blood pressure of the younger SHR animals (12 weeks), but none of the extracts were effective in the older SHR group (18 weeks). Vascular reactivity was also ameliorated with some of these treatments.

These extracts may be used as functional food ingredients with a moderate therapeutic benefit, especially in the early phases of arterial hypertension.

## Figures and Tables

**Table 1:** Main flavonoids of the products used in the present study.

Extract	Main Active Products
Apigenin	Apigenin: 93.7%; Other minor flavonoids (all): 3.5%
Lemon extract	Eriocitrin: 35.0%; Hesperidin: 1.5%; Other minor flavonoids (all): 3.5%
Grapefruit + bitter orange extract	Naringin: 54.3%; Neohesperidin: 10.9%; Other minor flavonoids (all): 4.8%
Cocoa extract	Epicatechin: 6.84%; Catechin: 0.86%; Procyanidins (dímers): 4.88%; Other flavan-3-ols (trimers to decamers, all): 18.42%
Diosmin	Diosmin: 93.20%; Other minor flavonoids (all): 4.7%

**Table 2:** Final mean arterial pressure (MAP) and heart rate (HR) in the experimental groups.

SHR6	MAP (mmHg)	HR (bpm)	SHR12	MAP (mmHg)	HR (bpm)
WKY	112.0 ± 3.7	363.6 ± 7.5	WKY	97.0 ± 7.1	313.6 ± 17.1
SHR	162.3 ± 5.7 *	384.9 ± 12.8	SHR	175.1 ± 5.4 *	395.3 ± 10.1 *
A	151.0 ± 6.9 *	379.2 ± 16.7	A	157.5 ± 9.2 *	399.8 ± 8.3 *
LE	152.7 ± 2.1 *	358.9 ± 10.8	LE	163.3 ± 5.9 *	369.2 ± 11.8 *
GBO	136.7 ± 6.3 *†	392.4 ± 12.9	GBO	167.8 ± 11.3 *	367.0 ± 5.6 *†
COE	153.3 ± 6.4 *	378.6 ± 12.9	COE	163.7 ± 9.4 *	363.8 ± 12.9 †
CPT	125.0 ± 1.8 *†	314.3 ± 9.1 *†	D	162.5 ± 5.8 *	356.4 ± 12.6 †

Abbreviations: WKY (Wistar Kyoto rats), SHR (spontaneously hypertensive rats), A (apigenin), LE (lemon extract), GBO (grapefruit + bitter orange extract), COE (cocoa extract), D (diosmin), CPT (captopril). Data are mean ± S.E.M. * *p* < 0.05 vs. WKY; † *p* < 0.05 vs. SHR.

**Table 3:** Metabolic values in the experimental groups.

SHR6	Body Weight (g)	Hematocrit (%)	Food Intake (g/24 h)	Water Intake (mL/24 h)	Diuresis (mL/24 h)	Natriuresis (mEq/24 h)	Sodium Balance (mEq/24 h/100 g)
WKY	317.2 ± 12.4	47.5 ± 0.6	20.9 ± 1.1	41.6 ± 4.4	11.2 ± 1.9	0.36 ± 0.03	0.57 ± 0.03
SHR	305.5 ± 4.7	55.0 ± 1.06 *	19.4 ± 0.4	28.4 ± 0,8 *	11.7 ± 0.9	0.41 ± 0.03	0.53 ± 0.02
A	304.8 ± 4.4	53.0 ± 0.5 *	22.4 ± 0 7 †	30.0 ± 1.7	10.6 ± 1.8	0.51 ± 0.06	0.6 ± 0.04
LE	309.2 ± 4.3	54.1 ± 0.8 *	18.2 ± 0.4 *†	28.8 ± 1.8 *	13.3 ± 1.3	0.71 ± 0.09 *†	0.38 ± 0.03 *†
GBO	302.4 ± 4.6	54.7 ± 1.3 *	19.8 ± 1.4	26.9 ± 0.9 *	9.7 ± 0.5	0.46 ± 0.05	0.53 ± 0.06
COE	302.9 ± 6.9	51.5 ± 0.7 *†	19.9 ± 0.7	33.1 ± 1.8	14.8 ± 1.7	0.57 ± 0.04	0.5 ± 0.03
CPT	322.2 ± 9.2	49.7 ± 1.9 †	18.5 ± 0.9	39.8 ± 4.2	20.5 ± 3.4 *	0.7 ± 0.14 *†	0.38 ± 0.04 *†
**SHR12**							
WKY	354.5 ± 7.7	45.5 ± 0.6	17.7 ± 0.8	33.9 ± 1.8	9.0 ± 1.2	0.15 ± 0.02	0.48 ± 0.02
SHR	359.1 ± 5.9	50.5 ± 0.9 *	18.2 ± 0.8	29.0 ± 1.6	11.8 ± 1.1	0.34 ± 0.04	0.41 ± 0.02
A	351.2 ± 8.7	52.2 ± 1.1 *	19.4 ± 0.8	28.3 ± 1.5	11.7 ± 0.9	0.53 ± 0.08	0.42 ± 0.03
LE	330.8 ± 11.8	49.5 ± 0.4 *	18.7 ± 0.6	28.9 ± 4.5	13.2 ± 3.4	0.35 ± 0.04	0.49 ± 0.04
GBO	342.4 ± 4.6 †	48.4 ± 0.4 *	20.1 ± 0.6 *	33.7 ± 2.0 *	12.6 ± 1.1	0.21 ± 0.04	0.55 ± 0.02 *†
COE	320.7 ± 16.3	50.4 ± 1.0 *	18.6 ± 0.7	25.7 ± 1.1	7.2 ± 1.0 †	0.19 ± 0.03	0.54 ± 0.02 *†
D	351.8 ± 8.0	50.6 ± 0.8 *	17.4 ± 0.5	20.6 ± 1.0	8.2 ± 0.8 †	0.36 ± 0.05	0.41 ± 0.02

Abbreviations: WKY (Wistar Kyoto rats), SHR (spontaneously hypertensive rats), A (apigenin), LE (lemon extract), GBO (grapefruit + bitter orange extract), COE (cocoa extract), D (diosmin), CPT (captopril). Data are mean ± S.E.M. * *p* < 0.05 vs. WKY; † *p* < 0.05 vs. SHR.

**Table 4:** Aortic vascular reactivity in the experimental groups.

	Phenylephrine		Acetylcholine		SNP
SHR6	pEC_50_ (mol/L)	Maximal Contraction (g)	Maximal Relaxation (%)	After Acute L-NAME	Maximal Relaxation (%)
WKY	−6.07 ± 0.05	2.71 ± 0.17	85.87 ± 2.67	34.17 ± 5.45	99.58 ± 0.99
SHR	−6.69 ± 0.11 *	2.13 ± 0.10 *	46.90 ± 3.72 *	3.70 ± 1.46 *	77.22 ± 3.19 *
A	−6.80 ± 0.07 *	1.94 ± 0.10 *	64.97 ± 1.63 *†	1.39 ± 2.76 *	88.43 ± 1.47 *†
LE	−6.60 ± 0.05 *	2.13 ± 0.11 *	55.97 ± 2.38 *†	3.47 ± 1.43 *	88.54 ± 1.86 *†
GBO	−6.60 ± 0.06 *	2.17 ± 0.13 *	60.56 ± 2.72 *†	1.56 ± 0.65 *	90.09 ± 2.18 *†
COE	−6.77 ± 0.03 *	2.13 ± 0.13 *	49.86 ± 4.86 *	0.86 ± 0.61 *†	91.07 ± 1.49 *†
CPT	−6.70 ± 0.07 *	1.46 ± 0.04 *†	95.57 ± 0.77 *†	19.43 ± 4.47 †	101.89 ± 1.34 †
**SHR12**					
WKY	−6.84 ± 0.06	2.62 ± 0.11	68.41 ± 2.80	11.26 ± 2.14	93.94 ± 2.21
SHR	−6.66 ± 0.05	2.03 ± 0.08 *	24.00 ± 2.68 *	1.47 ± 0.94 *	64.66 ± 3.04 *
A	−6.74 ± 0.05	2.25 ± 0.09 *	43.13 ± 3.45 *†	0.22 ± 0.23 *	80.28 ± 2.80 *†
LE	−6.75 ± 0.13	2.33 ± 0.15	26.50 ± 2.58 *	2.49 ± 1.07 *	70.64 ± 2.60 *†
GBO	−6.73 ± 0.1	2.27 ± 0.10 *	35.00 ± 8.44 *	2.45 ± 0.65 *	80.63 ± 5.48 *†
COE	−6.60 ± 0.11	1.94 ± 0.16 *	32.74 ± 6.39 *	3.07 ± 1.32 *	77.69 ± 5.82 *†
D	−6.68 ± 0.09	2.11 ± 0.14 *	21.38 ± 2.02 *	6.22 ± 2.24 *†	79.44 ± 3.78 *†

Abbreviations: WKY (Wistar Kyoto rats), SHR (spontaneously hypertensive rats), A (apigenin), LE (lemon extract), GBO (grapefruit + bitter orange extract), COE (cocoa extract), D (diosmin), CPT (captopril). Data are mean ± S.E.M. * *p* < 0.05 vs. WKY; † *p* < 0.05 vs. SHR. pEC_50_ is the negative logarithm of the half maximal effective concentration (EC_50_).

**Table 5:** Measurements of thiobarbituric acid reactive substances (TBARS), nitrite, and proteinuria in the experimental groups.

SHR6	Plasma TBARS (nmol/mL)	Kidney TBARS (nmol/mg prot)	Urine TBARS (nmol/mg prot 24 h)	Plasma Nitrite (µg/mL)	Urinary Excretion of Nitrite (µg/24 h)	Urinary Protein Excretion (mg/24 h/Kg bw)
WKY	6.3 ± 0.2	6.4 ± 0.4	252 ± 42	0.91 ± 0.02	11.6 ± 2.7	168.3 ± 50.4
SHR	7.6 ± 0.3 *	7.5 ± 0.4	559 ± 45 *	0.82 ± 0.02 *	13.9 ± 2.8	614.9 ± 28.8 *
A	11.8 ± 1.1 *†	6.7 ± 0.8	474 ± 35 *	0.95 ± 0.02 †	8.7 ± 1.8 †	563.6 ± 53.7 *
LE	10.2 ± 0.7 *†	5.8 ± 0.4 †	491 ± 37 *	0.89 ± 0.01 †	23.3 ± 5.3	540.7 ± 16.5 *†
GBO	7.2 ± 0.5	6.0 ± 0.3 †	335 ± 34 †	0.90 ± 0.03 †	15.7 ± 1.8 *	544.9 ± 26.2 *
COE	9.6 ± 1.5 *	4.4 ± 0.3 *†	473 ± 9 *	0.88 ± 0.03	23.3 ± 7.8	606.6 ± 50.7 *
CPT	6.0 ± 0.4 †	5.3 ± 0.4 †	615 ± 81 *	0.92 ± 0.03 †	23.7 ± 1.7 *†	551.6 ± 86.3 *
**SHR12**						
WKY	3.9 ± 0.4	8.3 ± 1.0	207 ± 22	1.13 ± 0.09	54.9 ± 11.2	213.3 ± 13.7
SHR	7.3 ± 0.4 *	11.1 ± 1.4	723 ± 32 *	1.01 ± 0.12	44.6 ± 3.3	547.5 ± 27.0 *
A	8.0 ± 0.4 *	6.9 ± 0.8 †	587 ± 17 *†	0.85 ± 0.02 *	37.6 ± 3.2	499.7 ± 25.2 *
LE	6.8 ± 1.2	7.3 ± 1.2	359 ± 27 *†	1.16 ± 0.02	44.7 ± 1.7	314.6 ± 47.1 †
GBO	6.7 ± 2.1	8.1 ± 0.4	336 ± 21 *†	1.28 ± 0.07	73.0 ± 10.6 †	306.4 ± 40.7 †
COE	4.6 ± 0.6 †	10.8 ± 2.5	331 ± 41 *†	1.28 ± 0.02	33.0 ± 4.3	353.1 ± 20.2 *†
D	3.1 ± 0.2 †	8.0 ± 0.6	339 ± 20 *†	1.36 ± 0.17	31.9 ± 2.8 †	451.0 ± 20.3 *†

Abbreviations: WKY (Wistar Kyoto rats), SHR (spontaneously hypertensive rats), A (apigenin), LE (lemon extract), GBO (grapefruit + bitter orange extract), COE (cocoa extract), D (diosmin), CPT (captopril). Data are mean ± S.E.M. * *p* < 0.05 vs. WKY; † *p* < 0.05 vs. SHR.

**Table 6:** Histopathological results of the heart, aorta, and kidney.

SHR6	Coronary LWR	Heart IVS	Thoracic AT	Abdominal AT	Renal LWR	Renal HA	Renal TC
WKY	2.27 ± 0.21	2.36 ± 0.14	107.2 ± 5.9	110.3 ± 7.5	1.69 ± 0.10	0.00	0.00
SHR	1.59 ± 0.03 *	2.95 ± 0.18 *	131.0 ± 5.6 *	138.8 ± 3.4 *	1.18 ± 0.08 *	0.00	0.00
A	2.84 ± 0.22 †	2.91 ± 0.13 *	125.3 ± 2.9 *	100.5 ± 2.1 †	1.32 ± 0.31	0.00	0.25
LE	1.80 ± 0.21	2.73 ± 0.05 *	150.4 ± 4.5 *	127.0 ± 3.6 †	1.51 ± 0.04 †	0.00	0.00
GBO	1.48 ± 0.13 *	2.50 ± 0.07	134.6 ± 2.3 *	117.5 ± 5.2 †	1.69 ± 0.10 †	0.00	0.00
COE	2.80 ± 0.58 †	2.83 ± 0.07 *	134.5 ± 3.8 *	87.1 ± 4.6 †	1.10 ± 0.24	0.00	0.00
CPT	1.78 ± 0.19	2.39 ± 0.12 †	133.3 ± 7.3*	101.7 ± 4.0 †	1.26 ± 0.10 *	0.00	0.00
**SHR12**							
WKY	3.08 ± 0.13	3.22 ± 0.15	104.8 ± 4.4	86.7 ± 5.8	1.76 ± 0.19	0.00	0.00
SHR	3.00 ± 0.80	3.43 ± 0.09	116.3 ± 2.2	90.5 ± 5.3	1.27 ± 0.26	0.25 ± 0.25	0.25 ± 0.25
A	1.97 ± 0.41	2.84 ± 0.23	126.8 ± 3.2 *†	94.6 ± 2.7	1.61 ± 0.30	0.00	0.75 ± 0.25
LE	3.43 ± 0.28	3.04 ± 0.29	137.5 ± 3.5 *†	114.8 ± 3.7 *†	1.67 ± 0.44	0.00	0.00
GBO	2.20 ± 0.44	3.01 ± 0.09	131.4 ± 3.6*†	107.6 ± 4.0 *†	1.79 ± 0.32	0.00	0.00
COE	3.06 ± 0.40	3.40 ± 0.15	142.2 ± 3.0 *†	118.8 ± 3.7 *†	1.39 ± 0.11	0.00	0.25 ± 0.25
D	3.15 ± 0.79	3.61 ± 0.09	130.8 ± 2.9 *†	104.6 ± 3.3 *†	1.43 ± 0.20	0.00	0.00

Abbreviations: WKY (Wistar Kyoto rats), SHR (spontaneously hypertensive rats), A (apigenin), LE (lemon extract), GBO (grapefruit + bitter orange extract), COE (cocoa extract), D (diosmin), CPT (captopril), LWR (lumen to wall ratio), IVS (interventricular septum width, mm), AT (aorta thickness, µm), HA (hyaline arteriopathy), TC (tubular cylinders). Data are mean ± S.E.M. * *p* < 0.05 vs. WKY; † *p* < 0.05 vs. SHR.
